# The interplay of galectins-1, -3, and -9 in the immune-inflammatory response underlying cardiovascular and metabolic disease

**DOI:** 10.1186/s12933-022-01690-7

**Published:** 2022-11-19

**Authors:** Adel Abo Mansour, Franziska Krautter, Zhaogong Zhi, Asif Jilani Iqbal, Carlota Recio

**Affiliations:** 1 Institute of Cardiovascular Sciences (ICVS), College of Medical and Dental Sciences, University of Birmingham, Birmingham, UK; 2 Department of Clinical Laboratory Sciences, College of Applied Medical Sciences, King Khalid University, Abha, Saudi Arabia; 3 Instituto Universitario de Investigaciones Biomédicas y Sanitarias (IUIBS), Farmacología Molecular y Traslacional -BIOPharm, Universidad de Las Palmas de Gran Canaria, Las Palmas de Gran Canaria, Las Palmas Spain

**Keywords:** Galectin, Inflammation, Immune system, Cardiovascular disease, Metabolic disorder

## Abstract

Galectins are β-galactoside-binding proteins that bind and crosslink molecules via their sugar moieties, forming signaling and adhesion networks involved in cellular communication, differentiation, migration, and survival. Galectins are expressed ubiquitously across immune cells, and their function varies with their tissue-specific and subcellular location. Particularly galectin-1, -3, and -9 are highly expressed by inflammatory cells and are involved in the modulation of several innate and adaptive immune responses. Modulation in the expression of these proteins accompany major processes in cardiovascular diseases and metabolic disorders, such as atherosclerosis, thrombosis, obesity, and diabetes, making them attractive therapeutic targets. In this review we consider the broad cellular activities ascribed to galectin-1, -3, and -9, highlighting those linked to the progression of different inflammatory driven pathologies in the context of cardiovascular and metabolic disease, to better understand their mechanism of action and provide new insights into the design of novel therapeutic strategies.

## Introduction

Galectins (Gals) are the most widely expressed class of lectins in all organisms. By binding carbohydrates via their sugar moieties, they form signaling and adhesion networks involved in a broad range of cellular responses including cell communication, activation, adhesion, migration, and apoptosis [1]. Gals are differentially expressed by various cell types, being immune and inflammatory cells one of the main Gal-expressing cells. According to this, some members of the Gal family are known to be master regulators of immune cell homeostasis and inflammation [1]. This fact makes them attractive molecular targets in different inflammatory-related diseases such as cardiovascular and metabolic pathologies [2, 3].

In the present review, we will dig into the role that these animal lectins exert in the inflammatory and immunoregulatory process in order to propose new strategies for the treatment of chronic inflammation-related pathologies. Interestingly, we will highlight how some members of the Gal family behave as amplifiers of the inflammatory cascade, whereas others restrain potentially damaging immune responses and suppress the spread of inflammation. All in all, we will summarise the consequences of these effects in the pathogenesis of cardiovascular and metabolic complications.

## Galectin family

Gals constitute a family of small soluble proteins that were originally defined based on their galactoside binding activity. They were found to share highly homologous (20–50%) conserved β-galactoside-binding sites within their carbohydrate recognition domains (CRDs) [4]. Interestingly, it was soon discovered that Gals were synthesised in the cellular cytosol and interact with their cognate galactoside ligands via non-classical secretion pathway [5, 6]. Gal nomenclature was derived in 1994, with Galectin-1 (Gal-1) the first identified by Teichberg and colleagues some years before [7]. The other members of the family were numbered consecutively by order of discovery [6]. Gals are characterised based on the presence of a conserved CRD required for glycan binding, the lack of calcium-dependence in glycan binding, and the use of an unconventional secretory pathway that has not yet been completely characterised [8]. To date, there are 16 Gals identified in mammals (from Gal-1 to Gal-16), of which 12 are human (Gal-5, -6, -11, -15, are not found in humans) [9].

**Fig. 1:**
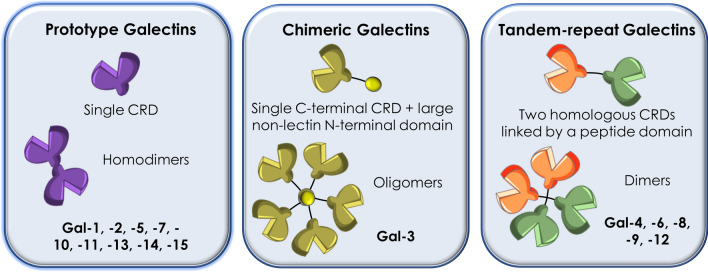
Classification of galectins according to structure

All Gals display at least one CRD in a single polypeptide chain that recognizes specific glycans on a variety of cell surface receptors and extracellular matrix. This CRD contains around 130 amino acids. However, only certain specific residues within the CRD directly bind glycan ligands [6]. A comparative study of more than 100 Gal sequences from many different sources revealed that there are eight invariant residues within the CRD that are involved in carbohydrate binding [6]. Additionally, there are several residues highly conserved between members of the family. Although most of Gals recognise simple β-galactosides, the binding affinity for such structures has been shown to be relatively weak [10]. Despite their high homology, Gals can be classified into three major groups according to their structure (Fig. 1):

- Prototype Gals, which contain a single CRD that may associate to form homodimers. This group includes Gal-1, -2, -5, -7, -10, -11, -13, -14, and -15. When they homodimerize, the two identical CRDs are held together by noncovalent electrostatic forces that are concentration dependent, without ligand influence [5, 10].
- Chimeric Gals, where Gal-3 is the only representative member, which is characterised by a single C-terminal CRD and a large non-lectin N-terminal domain (approximately 120 amino acids) rich in proline, glycine and tyrosine residues that may contribute to Gal-3 oligomerization [6, 10].
- Tandem-repeat Gals, characterised by containing two homologous CRDs (N-terminal and C-terminal) within a single polypeptide chain, linked by a small peptide domain (5 to 70 amino acids length). Gal-4, -6, -8, -9 and -12 belong to this group [6]

Ligands that bind Gals are essentially glycoproteins and glycolipids with different degrees of oligosaccharide modifications (N- and O-linked glycans). The selectivity and affinity that determines the binding of each Gal to a specific ligand is dependent upon the glycosylation sites within the sequence, the glycosylation levels in the Golgi complex processing, and the glycol-conjugate formations [6]. Thus, each individual Gal can preferentially interact with a set of glycoconjugates mediating specific functions, that usually differ from one Gal to another and also depending on the cell type and tissue [9].

Gals are found across all organisms, both vertebrates and invertebrates. Gal-like sequences have been detected not only in animals, but also in the genome of certain plants *(Arabidopsis thaliana).* Surprisingly, Gal-like protein expression has even been reported in some viruses such as porcine adenovirus and lymphocystis disease virus [6, 11].

Gals are widely expressed in many cell types, but their expression pattern varies between cell types and tissues [5]. Once synthesised on free polysomes in the cytoplasm, Gals can act both intra- or extracellularly. In the intracellular compartment, Gals bind to cytoplasmic and nuclear proteins in a carbohydrate-independent manner and regulate signal transduction and biological responses such as cell growth and apoptosis [6, 12]. Furthermore, Gals can interact with membrane components, and this interaction triggers the formation of vesicles involved in intracellular transport pathways, cellular degradation mechanisms and cell-to-cell communication signals [13]. In the extracellular milieu, Gals recognize specific oligosaccharides on cell surface glycoproteins and glycolipids or glycosylated extracellular matrix (ECM) and mediate biological signals that may modify cell interactions (cell–cell or cell–matrix) with and within the ECM [5].

The capacity of Gals to associate and form oligomers increases their glycan binding valency, leading to the formation of multivalent Gal-glycan complexes that can interact with several glycoconjugates simultaneously [14]. While prototype Gals can form homodimers, chimeric Gal-3 can form pentamers through its N-terminal domain, and tandem-repeat Gals can form oligomers [15]. Their organization into these multivalent complexes allows Gals to promote cross-linking, reorganization and clustering of glycosylated receptors thereafter modulating their activation and signaling [14, 16].

Gals regulate many crucial biological events including cell growth, apoptosis, pre-mRNA splicing, cell–cell and cell–matrix adhesion, cellular polarity, migration, differentiation, transformation, and signal transduction. Remarkably, some Gals display a key regulatory role in both innate and adaptive immunity [9].

## Galectins and the inflammatory response

The interplay between anti-inflammatory and pro-resolving mediators is an essential process for the resolution of immune responses. The lack of a precise regulation on their release and function may drive the setting of a chronic inflammatory state joined by collateral tissue damage and loss of immune cell homeostasis [17]. Some of the main mediators implicated in the resolution of inflammation include immunosuppressive cytokines, anti-inflammatory neuropeptides, bioactive lipid molecules, steroid hormones, and resolution-associated molecular patterns (RAMPs). Collectively these molecules neutralise the pro-inflammatory effects triggered by danger- and pathogen-associated molecular patterns (DAMPs and PAMPs, respectively) [18, 19].

**Fig. 2:**
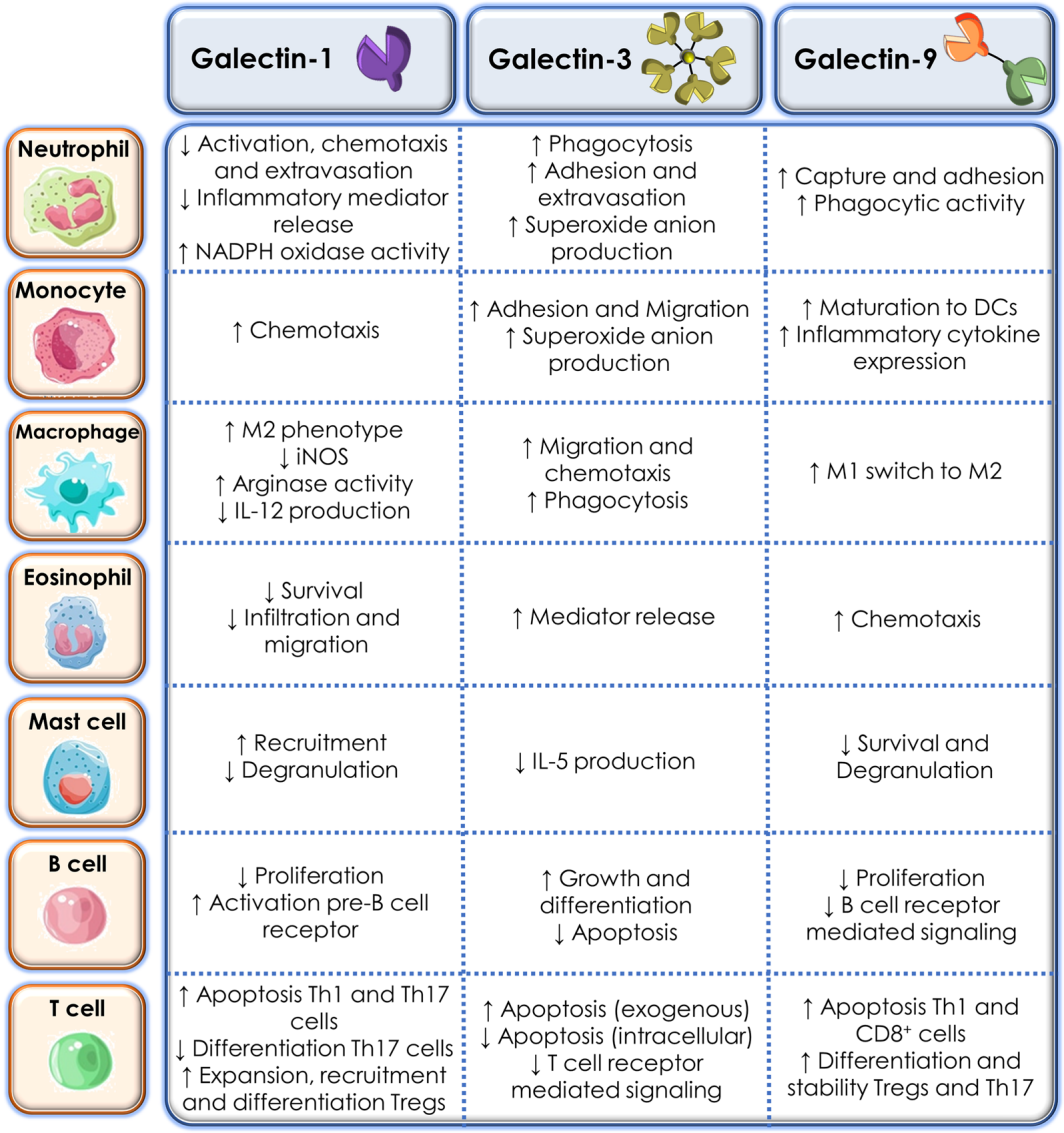
Role of Gal-1, Gal-3, and Gal-9 in innate and adaptive immunity [26–28]

Compelling evidence during the last decade have suggested that some members of the Gal family may act as RAMPs or DAMPs that function either in amplifying or resolving inflammatory responses [20]. Some of the main functions attributed to Gals during the inflammatory response is influencing the capacity of innate immune cells to respond to chemotactic gradients, migrate across the endothelium, synthesize and release pro- or anti-inflammatory cytokines, and recognize, engulf, and kill pathogens and/or damaged cells [21]. Regarding adaptive immunity, several Gals have been reported to influence T cell signalling and activation, module T cell survival, control the suppressive function of T regulatory (Treg) cells, alter cytokine profiles, and regulate B cell maturation and differentiation [22]. Recent review from Sanjurjo et al. proposed the concept of “galectokines” referring to heterodimers formed between Gals and cytokines that can either serve as a mechanism to stimulate or inhibit specific immune cell recruitment and functionality [23]. Several different galectokines have been reported, involving Gal-1, Gal-3, and Gal-9, and they appear to induce bidirectional effects between Gal and chemokine activities [24, 25]. Some Gals act primarily as pro-inflammatory mediators, whereas others display anti-inflammatory effects that trigger the resolution of inflammation; yet, in most cases, stimulatory or inhibitory actions vary depending upon the pathologic conditions, the tissue context, and the intra- or extracellular localization of the protein [14, 15] (Fig. 2).

### Galectin-1

Gal-1, composed of two subunits of 14.5 kDa, is synthesised and secreted by a wide range of immune cells, including activated macrophages, T and B cells, dendritic cells (DCs), and microglia, among others. Notably, organs with high immune activity such as thymus or lymph nodes, and those with immune privilege including placenta, testis, and eye, show prominent expression of Gal-1 [29–33]. Intracellularly, Gal-1 affects cell proliferation, cell cycle progression, and pre-RNA splicing, whereas in the extracellular milieu, it influences cell adhesion, aggregation, migration, and chemotaxis by binding to glycoconjugates on the cell surface [28]. Importantly, the oligomeric state of Gal-1 affects its function, as only dimeric Gal-1, but not its monomeric form, can induce phosphatidylserine exposure and enhance phagocytic recognition of leukocytes [34].

The large majority of data indicates an anti-inflammatory and pro-resolving role for Gal-1. This is exemplified through its ability to counteract pro-inflammatory cytokine synthesis, inhibit neutrophil trafficking, modulate monocyte and macrophage activation and support polarization towards an M2 phenotype, target eosinophil migration and survival, influence DC maturation and immunogenicity, and suppress mast cell degranulation [28, 35–37]*.* In addition, Gal-1 is known to regulate T and B cell activation, differentiation, and survival, acting as a negative-regulatory checkpoint on receptors required for signaling [38, 39] In particular, Gal-1 induces activated T cell apoptosis by binding CD45 receptor [2]. Interestingly, activated T cells can produce Gal-1 through MEK1/ERK, p38 MAP kinase signalling dependent pathway, suggesting that this is an autocrine suicide mechanism used to terminate an effector immune response [29]. Furthermore, it has been reported that Gal-1 is responsible for mitigating Th1- and Th17- mediated responses, thus driving the immune response towards a Th2-type profile [15, 40].

Numerous studies have described the potential of Gal-1 therapeutic applications in many disease settings. In experimental models of autoimmune disease including arthritis, diabetes, uveitis, and orchitis, Gal-1 elicits a wide range of immunoregulatory actions leading to the resolution of chronic inflammation [28, 41–44]. In contrast, Gal-1 has been shown to contribute to tumor evasion from the immune system by triggering apoptosis and preventing trans-endothelial migration of immune cells [39].

Considering the potential anti-inflammatory properties of Gal-1, the design of drugs based on its action should be imperative. However, its varying functionality and efficiency due to its different physicochemical states (monomer–dimer equilibrium and redox state), together with its conserved sequence similarity with other Gals, mean key challenges in drug development need to be overcome [39].

### Galectin-3

Notably, Gal-3 is the most extensively studied Gal in biomedicine. Its close relationship with the immune response has been widely reported in many different pathologic fields. This unique chimeric-type Gal of 30 kDa is broadly distributed throughout tissues, highlighting its expression in the digestive and urogenital tracts, lungs, blood, kidneys, and heart. Immune cells across the myeloid branch (monocytes, macrophages, DCs and neutrophils) also express high levels of Gal-3, as well as other cell types such as fibroblasts, epithelial and endothelial cells [45]. Gal-3 exerts its actions both intra- and extracellularly. Intracellular, it has been described to display antiapoptotic activity and regulate mRNA splicing, among other functions [46]. Conversely, Gal-3 extracellular effects are those more closely associated to the inflammatory response. Recent findings have demonstrated that Gal-3 can recognise PAMPs and promote the infiltration of leukocytes into the damaged tissues, being a key component in the host defense against infections [47] Gal-3 is known to drive neutrophil activation and adhesion, monocyte/macrophage chemoattraction, opsonization of apoptotic neutrophils, and activation of mast cells [48]*.* However, if tissue injury persists, Gal-3 appears to participate in the transition to chronic inflammation by displaying DAMP like properties through promoting wound healing and fibrogenesis which results in tissue architecture disruption and organ scarring [48].

Linking all the above-mentioned roles of Gal-3 in immunity with disease, it has been reported that Gal-3 plasma levels in cancer patients correlate with disease prognosis [2]*.* Additionally, Gal-3 has been reported to be actively involved in heart failure and associated pathologies. Indeed, its therapeutic potential in cardiomyopathy models is well described [49, 50]. Several studies have also shown the beneficial effects of Gal-3 inhibition in metabolic disorders [51, 52]. Remarkably, the most successful therapeutic application of Gal-3 to date is an inhibitor used on idiopathic pulmonary fibrosis treatment [53].

Noteworthy, Gal-3 is not the unique Gal with potential biomedical applications in the immune-inflammatory context; compelling evidence indicates both Gal-1 and Gal-9 are also key molecules involved in the regulation of the inflammatory response.

### Galectin-9

Gal-9 is a 36 kDa tandem-repeat galectin expressed in the liver, small intestine, and thymus, and to a lesser extent kidney, spleen, lung, and muscle [54]. Similarly to Gal-1 and -3, Gal-9 has attracted much attention in the last several years because of its compelling immunomodulatory effects [22]. Unlike other Gals that bind several receptors on immune cells, Gal-9 binds with higher affinity and specificity to the mucin domain-containing protein 3 (TIM3), a receptor expressed on T cells, monocytes, DCs and natural killer (NK) cells [55]. However, it has been reported that TIM3 may not be the only receptor by which Gal-9 signals, particularly in endothelial and epithelial cells [56]. Interestingly, in renal epithelial cells, Gal-9 has been shown to be an integral plasma membrane protein with at least two transmembrane domains, functioning as a highly specific urate transporter [27, 57]

In physiological conditions, Gal-9 has been described to be a pivotal modulator of T cell immunity by inducing apoptosis in specific T cell subpopulations [9, 56]. In the thymus, it participates in T cell selection by inducing apoptosis of CD4/CD8 double-negative or double-positive thymocytes, promotes naïve T cell differentiation into Tregs, and inhibits their differentiation into Th 17 cells [58, 59]*.* This ability of Gal-9 to promote apoptosis specifically in pathogenic Th1 and Th17 cells and promote resolution through Treg expansion, highlights its potential therapeutic exploitation in various autoimmune pathologies such as rheumatoid arthritis and systemic lupus erythematosus (SLE) which are known to be driven by T cells [58, 60, 61]. In fact, high expression levels of Gal-9 have been detected in rheumatoid arthritis synovial tissues and synovial fluid, where this lectin has been shown to inhibit the development of Th17 cells and increase the frequency of Tregs [27, 58]. Furthermore, in asthma, Gal-9 acts as an eosinophil chemoattractant and promoter of Th2 cells, thus contributing to the development of allergic airway inflammation [61].

Interestingly, the role of Gal-9 has also been widely explored in graft rejection. The inhibitory effect of this lectin on Th1 and T cytotoxic cell-mediated responses has been shown to prevent rejections induced by these cells, thus emerging as an attractive molecule in transplant therapy [62, 63].

In cancer, several studies have reported that Gal-9 is expressed by a variety of tumour cells and may play an important role in tumour immunity by regulating the survival, proliferation and migration of both tumour cells themselves and immune cells in the tumour microenvironment [37, 64].

Given the major activity of Gal-1, Gal-3, and Gal-9 in the inflammatory response, the remainder of this review will focus on considering these three Gals in different cardiovascular and metabolic driven diseases, in an attempt to enhance our understanding of their roles in the pathophysiology and to propose novel therapeutic strategies based on their action.

## Galectins in cardiovascular disease

Cardiovascular diseases represent one of the main causes of death worldwide. Among major risk factors, chronic low-grade inflammation has firmly been stablished as pivotal to the development and complications of several cardiovascular diseases [65]. Indeed, innate immune cells and inflammation are the trigger of the early stages of atherosclerosis, and they join disease progression to its thrombotic complications [66]. At the onset of atherosclerotic lesions, endothelial dysfunction and cholesterol accumulation triggers a subintimal inflammatory response [65]. A variety of adhesion molecules, cytokines, chemokines, and selectins promote the binding, rolling, and infiltration of inflammatory cells (monocytes and T cells) to early plaque initiation sites. These cells differentiate and release more inflammatory mediators that activate a higher number of inflammatory cells thus amplifying the inflammatory cascade within the vessel wall [65, 66]. The non-resolution of the inflammatory burden may trigger atherosclerotic plaque progression and rupture, thereby leading to thrombotic complications and ultimately provoking fatal cardiovascular events [67].

It has been reported that elevated levels of inflammatory markers (C‑reactive protein, cytokines) are associated with a higher risk of developing both cardiac and vascular events and worse survival, and several studies have shown that targeting immune-inflammatory function in experimental models can attenuate disease progression and promote healing [67–70]With this in mind, this review will focus on the role that Gals, as important immunoregulatory proteins, play in the inflammatory response underlying cardiovascular diseases, and will highlight the potential of targeting each individual Gal on each pathology.

## Atherosclerosis

Atherosclerosis is an inflammatory disease of large and medium-sized arteries, manifesting itself in, for example, peripheral or coronary arterial disease (PAD and CAD, respectively). Symptoms such as claudication occurs only after many decades of plaque formation and progression in the arterial wall [71]. Over the years, plaques progress from fatty streaks to a more fibrous rich,—necrotic core containing structure. The whole process is driven by various cell types: monocytes initially transmigrate through endothelial cells which are activated by lipid depositions and disturbed blood flow [72]. These monocytes differentiate into macrophages and foam cells, taking up the lipids and releasing pro-inflammatory mediators [73]. This induces vascular smooth muscle cells (VSMCs) migration which leads to the formation of the fibrous cap through their release of components such as elastin and collagen [74]. The expansion of the necrotic core through continuous influx of leukocytes attracted by the pro-inflammatory stimuli leads to decreased blood flow and ischemic events, downstream of the plaque [72]. If the fibrous cap thins, and the necrotic core is exposed, thrombogenic agents are released, leading to thrombus formation, potentially causing strokes or heart attacks [75].

The role of Gals in various stages of atherosclerotic disease and as biomarkers have been assessed in a range of studies.

### Galectin-1

Gal-1 serum levels have been found upregulated in patients with larger artery atherosclerotic strokes at day 1 and 6 as well as 4 weeks post stroke and in age and sex-matched controls [76]. In this study, He et al*.* found that Gal-1 levels were upregulated 4 weeks after the stroke occurred, compared to the healthy controls, suggesting that Gal-1 serum levels might be associated with large artery atherosclerotic stroke. Gal-1 levels in plaques however did not change with increasing inflammation, when they compared Gal-1 levels using immunohistochemistry in plaques of ApoE^−/−^ mice on high fat diet for either 16 or 26 weeks [77, 89]. Furthermore, another study showed that Gal-1 increased VSMC binding to ECM components by strengthening the integrin-ECM interaction and thereby decreasing VSCM motility [78]. Since VSMC motility is an important part of atherogenesis, Gal-1 might play a role in this pathological process.

### Galectin-3

Numerous studies have also shown that Gal-3 levels in serum or plasma are increased as a result of peripheral or coronary artery disease, and large artery atherosclerotic stroke as well as diet-induced atherosclerosis in ApoE^−/−^ mice [76, 77, 79–84]. Many of these studies indicate a positive correlation between Gal-3 levels in serum or plasma and the severity of the disease or inflammation markers such as CCL2 or CRP [81, 85]. Other studies looked at Gal-3 binding protein which was also increased in plasma levels of coronary artery disease patients and was associated with long-term mortality [86]. Investigations of Gal-3 binding protein levels as part of a 4-biomarker signature in vascular extracellular matrix of atherosclerosis patients showed a positive correlation of ECM levels with atherosclerosis progression and incidences of cardiovascular diseases [87]. These studies all highlight the use of Gal-3 and Gal-3-binding protein as biomarkers for (the severity of) arterial disease. Especially since Gal-3 also can be used to differentiate inflammatory cardiovascular disease from other inflammatory diseases such as diabetes or rheumatoid arthritis [80, 84]. However, it must be used in combination with other biomarkers to make reliable predictions and a standardised ELISA test has to be established since the detected levels vary greatly (between average values of 4.5–17.6 ng/ml in arterial disease patients and 2.8–14.4 ng/ml in healthy controls) [88, 89].

In vivo studies using ApoE^−/−^ xGal-3^−/−^ mice showed that these mice had significantly lower numbers of atherosclerotic lesions and atheromatous plaques as well as smaller plaques with a smaller lipid core and less collagen compared to ApoE^−/−^ mice, highlighting a role for Gal-3 in atherogenesis and not just as biomarker [90, 91]. Additional studies have demonstrated two separate pathways in which Gal-3 enhances the activation of endothelial cells by oxidised low density lipoprotein (oxLDL). It was demonstrated that Gal-3 increases oxLDL-mediated upregulation of inflammatory markers Il-1ß, IL-6, IL-8, CXCL-1, CCL2, ICAM-1 and VCAM-1 on HUVECs through a ß1-integrin-RhoA-JNK mediated pathway [92]. On the other hand, a different pathway leading to increased inflammation of HUVECs after oxLDL-mediated inflammation has been reported. The authors found that Gal-3 promoted inflammation by upregulating LOX-1, an oxLDL receptor, which mediates a ROS/p38/NFkB-mediated signalling pathway, leading to increased adhesion molecule expression and resulting in increased adhesion of monocytic cells as well as IL-8 secretion [93]. Whether the different pathways leading to enhanced activation of oxLDL-stimulated HUVECs occur due to the different amounts of Gal-3 (250 ng/ml and 2.5–20 µg/ml or different sources of the oxLDL used needs to be further established [92, 93].

Increasing adhesion of monocytic cells by activating the endothelium is not the only way in which Gal-3 contributes to monocyte-mediated atherogenesis [93]. An earlier study demonstrated that Gal-3 induces monocyte and macrophage migration in a concentration dependent manner [94]. Lee et al*.* initially showed a positive correlation between macrophage content and Gal-3 content of plaques in ApoE^−/−^ mice on high fat diet [77]. Madrigal-Matute et al. furthered this and identified phorbol myristate acetate (PMA)-activated monocytes and macrophages as source of Gal-3 which was released through a pathway involving exosomes [85]. Furthermore, Di Gregorli et al. have recently shown that Gal-3 marks a macrophage subset in atherosclerotic plaques with potentially beneficial characteristics by regulating macrophage polarisation as well as invasiveness which leads to a slower plaque progression [95]. Further research is required to investigate how Gal-3 can act as pro- and anti-atherogenic factor.

**Fig. 3:**
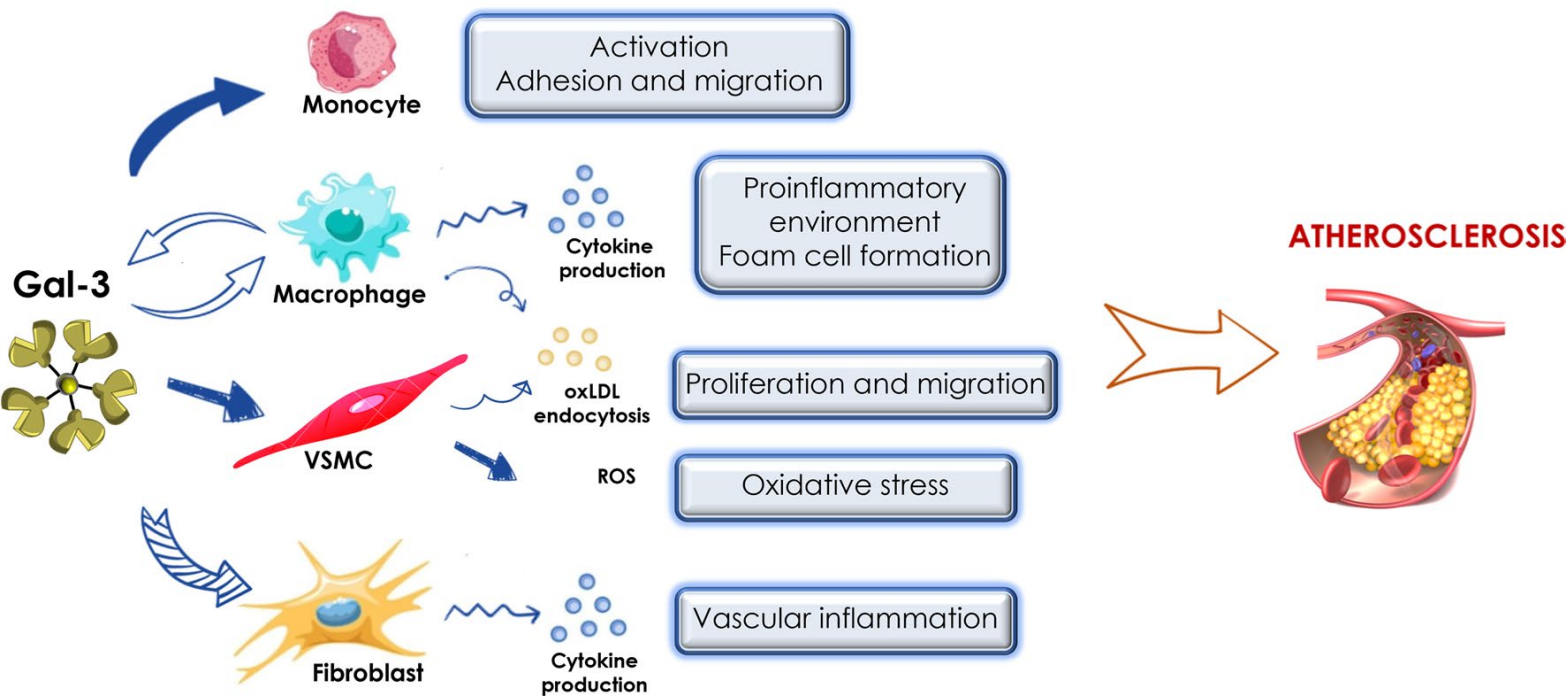
Gal-3 in atherosclerosis

The phenotypic change of vascular smooth muscle cells as well as their migration marks atherosclerotic progression (Fig. 3). It was shown that the Gal-3 expression patterns in the arterial wall differed between PAD patients and healthy controls: in healthy controls, Gal-3 was mainly in the adventitia whereas in PAD patients, Gal-3 was mainly found in the media, adjacent to SMCs [82]. Another study further highlighted the role of Gal-3 on VSMCs in atheroprogression. Using Gal-3 knockdown in SMCs, authors showed that endogenous Gal-3 expression in VSMCs upon oxLDL stimulation is essential for the phenotypic change marked by increased osteoponin, calponin and alpha-actin expression leading to increased migration, proliferation as well as phagocytosis [96]. They suggested that a canonical Wnt/ß-catenin signalling pathway is responsible for this Gal-3 mediated phenotypic shift. The same group further expanded the understanding of how Gal-3 is involved in SMC regulation in a follow up study. They showed that exogenous Gal-3 also promoted human umbilical vascular smooth muscle cell (HUSMC) proliferation and migration as well as the proteins marking the phenotypic changes in their previous study. Again, they demonstrated that these changes are triggered through a Wnt/ß-catenin signalling pathway [97]. Another study showed that the same pathway is involved in Gal-3/RAGE mediated calcification patterns of VSMCs [98]. These findings were further expanded by a study in the commonly applied atherosclerotic in vivo model using ApoE^−/−^ mice. It was shown that Gal-3 and RAGE regulate sortilin in opposite ways, resulting in up- and downregulation respectively. This consequently led to different calcification patterns where RAGE caused microcalcification and Gal-3 caused macrocalcification of atherosclerotic plaques in the ApoE^−/−^ mice [99].

Collectively these studies show that Gal-3 contributes in multiple ways to atheroprogression but also its ability to act as beneficial regulator in macrophages, highlighting the complex nature of Gal-3 activity. To investigate its potential as therapeutic target, several studies used modified citrus pectin (MCP), a carbohydrate known to bind Gal-3. Treatment of ApoE^−/−^ mice on high fat diet with MCP i.v., was found to decrease plaque volume, atherosclerotic lesion numbers as well as macrophage and smooth muscle cell numbers [91, 100]. It was also shown that MCP treatment reduced the adhesion of monocyte to oxLDL stimulated endothelium [100]. The short-term treatment of ApoE^−/−^ mice on high fat diet with atorvastatin, a statin, decreased Gal-3 mRNA and protein levels in aortic plaques [77]. However, another study in patients with carotid artery atherosclerosis showed that long term treatment with statins increased Gal-3levels but decreased macrophage numbers inside of the plaques [101]. They concluded that this may mediate plaque stabilisation. These studies clearly highlight the therapeutic potential of Gal-3, either as therapeutic or as a target. However, further studies are needed to understand the complex mechanisms in which it acts in atherogenesis to be able to use it as successful therapeutic target.

### Galectin-9

Regarding Gal-9, only two studies have analysed its role in context of atherosclerosis to date. A study reported increased serum levels of Gal-9 in patients with large artery atherosclerotic stroke [76]. Interestingly, a study which measured Gal-9 serum levels in coronary artery disease patients found decreased levels compared to healthy controls. The study also evaluated the effects of Gal-9 on T cells. They reported that Gal-9 shifted T cell phenotypes towards Tregs, while supressing Th 17 cells, therefore decreasing IL-17 production [102]. Furthermore, a study investigating the effects of Gals on leukocyte adhesion found that the incubation of monocytes with Gal-9 increased their adhesion to HUVECs [103]. However, the transmigration of monocytes was not quantified and whether the increased adhesion may contribute to increased plaque formation needs to be further established.

## Myocarditis

Myocarditis is a life-threatening inflammation of cardiomyocytes. It is caused by sterile triggers such as autoimmune disease, side effects of drugs or viral and parasitic infections such as *T. cruzi*. The acute phase of inflammation is the direct response to these triggers, and if unresolved, leads to the chronic phase, marked by fibrosis. In severe cases, this can lead to heart failure and death [104].

The various roles of Gals in myocarditis have been investigated in a series of studies.

### Galectin-1

Increased Gal-1 has been found in serum of asymptomatic and symptomatic patients in the chronic stage of Chagas Disease as well as cardiac tissue [105, 106]. Studies have shown that *T.cruzi* infected macrophages as well as murine cardiac cells (HL-1 cell line) release Gal-1 alongside the cytoplasmic enzyme lactate dehydrogenase upon *T.cruzi* infection, suggesting that the macrophages and the lysis of the cardiac cells might be sources of Gal-1 in the serum and possibly heart tissue. Increased anti-Gal-1 autoantibodies were found in serum of chronic Chagas Disease patients, possibly due to an autoimmune response against the released Gal-1 from the lysed cardiac cells, similar to previously reported autoimmune responses in cardiomyopathies against myosin, actin or myoglobin [105–108]. Furthermore, they demonstrated in vitro and in vivo, that Gal-1 blocks infection of cardiac cells in a glycan-dependent manner. However, these findings are gender- and *T.cruzi* strain-dependent. Other studies have shown a concentration dependent mechanism in which Gal-1 acts in *T.cruzi* infections. Zúñiga et al. showed that low concentrations of Gal-1 increased parasite replication and downregulated anti-parasitic agents such as IL-12 whereas high concentrations induced apoptosis and prevented parasite replication [107]. Whether this model also replicates what happens in cardiac tissue with increased levels of Gal-1 due to *T.cruzi* infection needs further investigation. Further studies are also needed to be able to understand possible effects of Gal-1 on fibrosis and autoimmune myocarditis.

### Galectin-3

By depleting CVB3-infected mice of macrophages (liposome encapsulated clodonate), it was identified that macrophages are the main source of Gal-3 during acute and chronic phases of myocarditis [50]. These findings are in line with findings from other studies where Gal-3-positive cells were identified as macrophages during myocarditis [50, 109, 110].

Studies looking at viral induced myocarditis have described a pro-inflammatory role of Gal-3. It has been reported that Gal-3 positive cells (activated macrophages and DCs) infiltrate the heart and that Gal-3 levels correlate with fibrosis or that Gal-3 increased fibrosis [109–111]. Several studies have confirmed these findings in in vivo models: decreases in infiltration of the tissue by leukocytes, inflammatory markers (CCL-2), and fibrosis (proCol I mRNA, alpha Smooth Muscle Actin) were observed in Gal-3 knock-out models [50, 112, 113]. These studies all highlight the role of Gal-3 as pro-inflammatory agent in viral myocarditis. The potential of Gal-3 as therapeutic target was highlighted, when it was shown that pharmaceutical inhibitors such as N-Lac were able to decrease inflammation by targeting Gal-3 [50, 110]. However, even though a study by Souza et al*.* could demonstrate decreased fibrosis in in vitro when Gal-3 was knocked-down, they could not report any improvements in frequency or severity of arrhythmias when Gal-3 was blocked [110]. Other authors showed less fibrosis in the chronic phase when CVB3-infected mice were treated with N-Lac [50]. These findings confirm the decreased numbers of inflammatory cells infiltrating the heart and less fibrosis during chronic inflammation caused by *T. cruzi* in mice treated with N-Lac [110]. Similar trends were also shown in myocarditis caused by increased aldosterone. Decreased inflammation and lower fibrosis was found when Gal-3 was inhibited with citric pectin in rats [113]. It was also shown that aldosterone upregulates Gal-3 expression in cardiac fibroblasts and suggest that Gal-3 could be a novel molecular mechanism linking high aldosterone levels with increases in inflammation and fibrosis [113]. In summary, Gal-3 appears to have a pro-inflammatory effect in the acute and chronic phases of pathogen- induced myocarditis.

Interestingly, Gal-3 appears to have an anti-inflammatory effect in experimental autoimmune myocarditis (EAM) compared to a pro-inflammatory effect in virally induced myocarditis. In fact, increased EAM has been demonstrated in Gal-3 KO mice, accompanied by increased infiltration of CD45 + leukocytes into the heart [114]. This study suggested that Th2 cells play a major role in the increased inflammatory response but also mention that the increased IgG might cause damage to the myocytes by triggering antibody-dependent cell mediated cytotoxicity since Gal-3 inhibits plasma cell differentiation and lack of Gal-3 favours antigen-specific antibody production.

### Galectin-9

A role of Gal-9 in myocarditis has also been reported. It was shown that Gal-9 mRNA and protein levels are increased in heart tissue and serum in CVB3 infected mice between day 3 and 14 post infection, peaking on day 7, however, the source of Gal-9 remains to be determined [115]. Additionally, authors were able to show that the administration of Gal-9 to CVB3 infected mice showed reduced CD4 + T-cells, but increased Treg in the heart tissue 7 days post infection, which was complimented by decreased Th1 cell cytokines (TNF, IFN), but increased Th2 cytokines (IL-4, IL-10) [115]. Zhang et al*.* confirmed these findings in their study adding an increase of Treg cells also in the spleen after mice received the same treatment [116]. They showed that the expansion is due to CD11b + Gr1 + myeloid -derived suppressor cells which are more frequent in Gal-9 treated mice infected with CVB3 then in the PBS-treated control mice. In a follow up study, they were able to expand further and show that a Ly6C + subset of these suppressor cells is causing the increase of Tregs and Th2 cell frequency. This increase is accompanied by an increase in anti-inflammatory cytokine expression in heart tissue as well as a decreased CVB3-induced myocarditis in mice upon Gal-9 treatment [117]. Further studies need to be conducted to identify the source of Gal-9 during myocarditis and to show the effects of Gal-9 administration on other cell types involved in acute inflammation. Also, long term studies on the role of Gal-9 in fibrosis need to be conducted to eventually determine the true therapeutic potential of Gal-9.

## Thrombosis

**Fig. 4:**
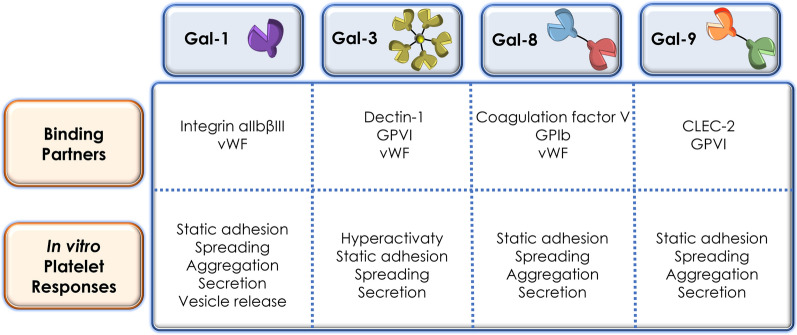
Galectins in thrombosis and platelet activation [121–124, 127, 129–133]

Thrombosis, the excessive haemostatic process in response to certain pathological conditions, represents a critical pathogenic mechanism inducing numerous fatal vascular diseases, such as Acute Coronary Syndrome (ACS), stroke and Venous Thromboembolism (VTE) [118]. Emerging evidence has demonstrated that platelet activation, as a major process involved in thrombosis and thrombo-inflammation [119, 120], can be induced by various Gals (Fig. 4).

### Galectin-1

Gal-1 is expressed on the extracellular surface of human platelets [121]. Gonzalez et al. reported that Gal-1 forms complexes with monomeric actin intracellularly and co-purifies with actin in human platelets [122]. ADP-induced platelet aggregation can be inhibited by lactose or anti-Gal-1 serum in vitro [121]. Interestingly, washed platelets from Gal-1-deficient mice showed similar aggregation response to arachidonic acid, ADP, collagen, and protease-activated receptor 4–activating peptide (PAR4-AP) in comparison with the WT group, but exhibited impaired adhesion to fibrinogen-coated surface under static condition and delayed clot retraction [123].

Exogenous Gal-1 has been shown to be a potent agonist that induces a broad range of platelet functions in a carbohydrate-dependent manner, with the α_IIb_ subunit of α_IIb_β_3_ integrin as a significant binding partner, which implies that exogeneous Gal-1 may come into different play in platelet activation from endogenous Gal-1 [123]. Similar to traditional platelet agonists, Gal-1 is capable of inducing aggregation and degranulation in human washed platelets, such as the release of ATP, platelet factor 4 and platelet-derived proangiogenic factors, and P-selectin expression [121, 123, 124]. However, as reported by Dickhou et al., Gal-1 failed to induce P-selectin expression without neuraminidase treatment or phosphatidylserine exposure in human platelets in vitro [125]. Immobilised Gal-1 efficiently mediates the adhesion of human washed platelets under static condition and induces F-actin polymerization and subsequent extensions of filopodia and lamellipodia [121, 123, 125]. Furthermore, Gal-1 not only directly triggers platelet aggregation, but also synergizes with ADP and thrombin enhancing their effects on platelet aggregation in vitro [121]. The study of Pacienza et al. also found that Gal-1 enhanced shedding of platelet-derived extracellular vesicles (PEVs) carrying an abundance of P-selectin and induced heterotypic leukocyte-platelet aggregation in vitro [121]. The interaction between P-selectin to P-selectin glycoprotein ligand has been well-known to support platelet-mediated leukocyte recruitment [126]. Hence, the action of Gal-1 in platelet activation could be a novel angle to appreciate its immunoregulatory activities. To note that the evidence of Gal-1 in the context of platelet activation and thrombosis are mostly collected in vitro, apart from the prolonged tail bleeding time in Gal-1-deficient mice [123]. The doses of recombinant Gal-1 utilised in above studies ranges from 1 to 12 µM, which could already be excessive considering the concentration of Gal-1 in vivo. Therefore, whether Gal-1 truly plays a role in haemostasis or thrombosis in vivo remains further investigation.

### Galectin-3

Gal-3 has been recently identified as a ligand for glycoprotein VI (GPVI) on platelets [127]. Murine platelets adhere and spread on immobilised Gal-3 under static condition in vitro*.* Meanwhile, tumour cell (MC38)-derived Gal-3 modulates P-selectin expression and ATP release of murine platelets via GPVI [127]. The authors demonstrated that the interaction between Gal-3 and GPVI promoted metastasis of colon and breast cancer by triggering ATP release of platelets, and subsequently enhancing permeability of vascular endothelium and extravasation of tumour cells [127, 128]. However, whether Gal-3 directly participates in thrombosis remains unclear. More recently, Chen et al*.* reported that Gal-3 at plasma level in patients with coronary artery disease primed platelets into hyperactive state via interacting with Dectin-1 and enhanced their responses to traditional agonists ADP, collagen and thrombin, and thrombus formation on immobilised collagen in vitro [129]. Intravenous administration of Gal-3 in FeCl_3_-induced thrombosis model accelerated initial thrombus event and increased thrombus area in WT mice but failed to make difference in Dectin-1^−/−^ deficient mice or thrombocytopenic WT mice transfused with Dectin-1^−/−^ platelets [129].

Saint-Lu et al. demonstrated that Gal-1 and Gal-3 formed complexes with von Willebrand Factor (vWF) carbohydrate-dependently in both circulation and endothelial cells. Plasma levels of Gal-1 and Gal-3 in WF-deficient mice were also found significantly lower than WT controls [130]. Importantly, Gal-1 and Gal-3 exhibited an inhibitory effect on modulating vWF-string formation and platelet-vWF interaction. FeCl_3_-induced thrombosis model in WT and Gal-1^−/−^/Gal-3^−/−^ mice showed that the initial thrombus events in the Gal-1^−/−^/Gal-3^−/−^ group occurred more rapidly than the WT controls, while the time to first occlusion events remained no difference between Gal-1^−/−^/Gal-3^−/−^ and WT groups, which indicates that thrombus growth was comparatively faster in the WT group [130]. This observation suggested that the absence of Gal-1 and Gal-3 contributed to the early stage of thrombosis but slowed down the thrombus growth. Of note, the binding of vWF to glycoprotein Ib (GPIb) and collagen was not interfered by its interaction with Gal-1 and Gal-3. The mechanism how Gal-1 and Gal-3 inhibited early arterial thrombus formation remains to be investigated.

### Galectin-8

Gal-8 is the first tandem-repeat galectin identified as a platelet agonist, modulating static adhesion, spreading, aggregation and degranulation of human washed platelets in vitro*,* with a putative receptor GPIb [131]. Rumaniuk et al*.* also proposed that bivalency of Gal-8 is not essential for Gal-8 induced platelet activation as only the recombinant Gal-8N-CRD activated platelets rather than the C-CRD.

### Galectin-9

Recently, our group reported that Gal-9, as a novel ligand of platelet receptors GPVI and CLEC-2, induces aggregation, adhesion, spreading and secretion in both human and murine platelets in vitro [15]. The binding between Gal-9 and GPVI tended to be a protein–protein interaction and the binding site of Gal-9 on GPVI was found overlapped with the collagen-binding site [15].

## Myocardial infarction

Myocardial infarction (MI) is pathophysiologically characterised as ischemic myocardial injury caused by prolonged imbalance between myocardial oxygen delivery and consumption [134]. The most common aetiology of this fatal cardiovascular event is an acute and severe reduction in the blood flow of culprit coronary arteries, as a result of atherosclerosis with or without atherothrombosis, which is categorised as Type 1 MI [135].

**Fig. 5:**
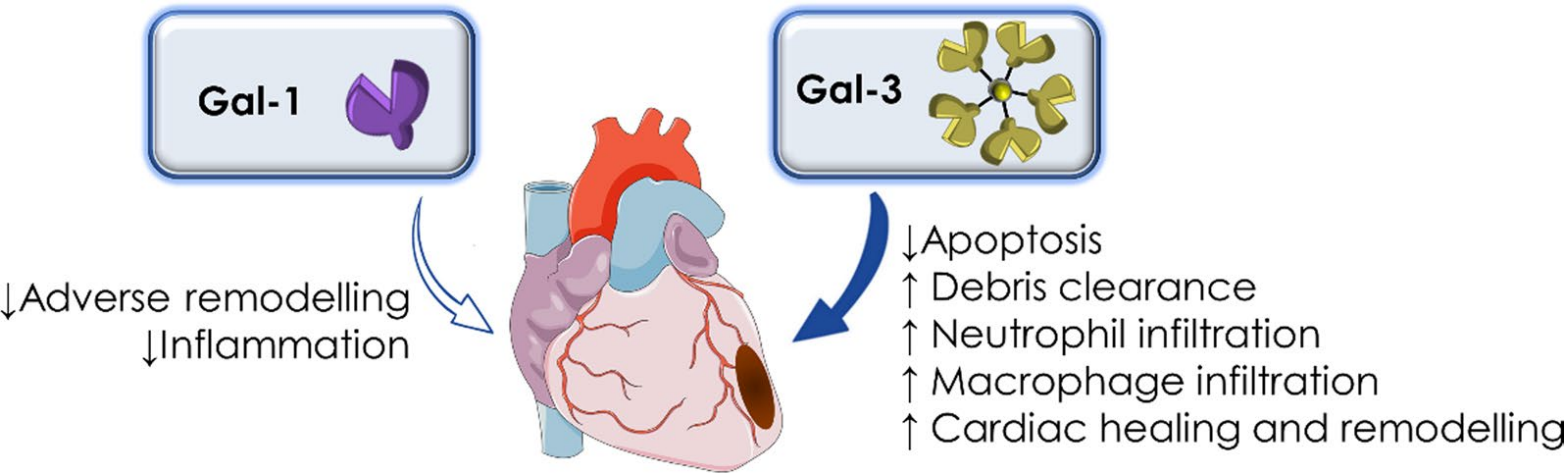
Gal-3 and Gal-1 in MI

Within the Gal family, Gal-3 bears the most evidence and a leading role in MI. Circulating levels of Gal-3 have been proposed as a biomarker of heart failure by American Heart Association, with promising potential as a predictor of clinical outcomes concerning Acute Coronary Syndrome in various clinical studies [136, 137]. Conversely, the role of Gal-1 and -9 in MI has not drawn as intensive attention as that of Gal-3. In this section, we mainly focus on the experimental findings of Gal-1 and Gal-3 (Fig. 5).

### Galectin-1

Gal-1 is expressed by murine and porcine cardiomyocytes during normal physiology [138, 139]. Interestingly, in human cardiac specimens retrieved from non-cardiac death autopsy cases, expression of Gal-1 was reported to be scarcely detected or even undetectable via histologic assay [140]. Although the number of cases included in the study is very restricted, this finding might imply different levels of Gal-1 expression in normal hearts between human and other mammalian species. However, Gal-1 was found significantly upregulated in cardiomyocytes of patients with end-stage heart failure regardless of aetiology [140]. In striated muscle, the intracellular spatial distribution of Gal-1 appeals to be an organised and striated pattern colocalising on I band with sarcomeric actin instead of myosin [138, 139]. Accordingly, endogenous Gal-1 seems to participate in the mediation of cardiomyocyte contractility, which requires further investigation.

Apart from the characteristic spatial expression, the presence of Gal-1 in infarcted myocardium significantly upregulates and represents a bimodal temporal distribution in murine model. Experimentally, the expression of Gal-1 in left ventricular myocardium during MI reaches its first peak at 20 min after left anterior descending (LAD) artery ligation, while the second peak is present round 7 days later [139, 140]. The upregulation of Gal-1 is predominantly restricted to the myocardial area supplied by ligated artery. During the first four hours following ligation, cardiomyocytes, and cardiac endothelial cells (ECs) are two dominant groups presenting Gal-1. As ischemia goes on and the scale of cell death in both group increases, focal areas with low or without Gal-1 expression become increasingly evident after 4-h ligation. At 24 h after LAD ligation, infiltrated neutrophils are also observed presenting Gal-1 in addition to cardiomyocytes and cardiac ECs. Cardiac expression of Gal-1 is primarily localised in periphery of infarcted region [139]. On the 7th day after coronary ligation, the level of Gal-1, which is mostly expressed by cardiomyocytes, infiltrated mononuclear cells and polymorphonuclear neutrophils in left ventricle, peaked at about 7 times higher than the level at 24-h ligation [140].

Hypoxia has been shown to be a key factor driving the first peak of Gal-1 expression in infarcted region [140]. Seropian et al. showed in vitro enhanced Gal-1 expression of cultured HL-1 cardiomyocytes in response to experimental hypoxia [140]. Furthermore, HIF-1 has been illustrated to induce Gal-1 upregulation in four representative lines of colorectal cancer cells [141]. The study of Al-Salam et al. observed a simultaneous peak of HIF-1 expression at 20 min following LAD ligation in cardiac specimens and colocalised HIF-1 and Gal-1 in both cardiomyocytes and cardiac ECs, which were the main source of Gal-1 expression in infarcted area during the initial 4 h of MI [139]. The second peak of Gal-1 upregulation is in line with enhanced infiltration of DCs, lymphocytes, and macrophages in infarcted myocardium. It was also found that Gal-1^−/−^ mice endured more significant adverse LV remodelling post MI and inflammatory cell infiltration in myocardium, indicating the potential of Gal-1 to attenuate adverse remodelling and inflammation during MI but the underlying mechanism needs further exploration [140].

### Galectin-3

In normal murine heart, both Gal-1 and Gal-3 were reported to be more markedly expressed in the right ventricle [139, 142]. Cardiac ECs account the major source of Gal-3 expression in normal myocardium, whilst cardiomyocytes or cells in interstitial tissue are seldomly found presenting Gal-3 [142, 143]. By contrast, Gal-3 is highly upregulated in cardiomyocytes and cardiac ECs in murine MI models [142, 144]. The presence of Gal-3 in cardiomyocytes within the infarcted region shows a I-band staining pattern colocalising with desmin histologically, which is similar to the distribution of Gal-1 in cardiomyocytes [142]. In infarcted region, Gal-3 as a DAMP can be released by necrotic ECs and cardiomyocytes [45, 145–147]. In addition, Gal-3 expression was observed in murine neutrophils infiltrated in infarcted myocardium and cultured cardiac fibroblasts [142, 148].

Gal-3 in the infarcted region exhibited persistent upregulation and reached its peak after 7 to 14 days in non-reperfused murine model according to different studies [149–152]. The overall expression of Gal-3 in the entire LV increased significantly at mRNA and protein level following 30 min after ligation, and the infarcted area showed demarcated and more prominent Gal-3 expression than the non-infarcted region (remote zone) [142]. At 24 h post-MI, highly expressed Gal-3 in myocardium mainly concentrates in the peri-infarcted region (also termed as border zone), whilst infiltrated neutrophils consist in the only dominant group with Gal-3 expression in the infarcted area since the loss of surviving cardiomyocytes and cardiac ECs [142]. The study of Al-Salam et al*.* detected increased apoptotic bodies and cells, significantly downregulated pro-apoptotic proteins, and upregulated anti-apoptotic proteins in myocardium, as well as enhanced lysosomal activity in surviving cardiomyocytes of Gal-3-deficient mice at 24 h post-MI [144]. Hence, it was proposed that endogenous Gal-3, the lectin which contained an Asp-Trp-Gly-Arg (NWGR) anti-death motif, exhibited an anti-apoptotic activity in myocardium, especially for cardiomyocytes [144]. During the later time course of MI, increasingly infiltrated macrophages were considered as another major source of Gal-3 in the infarcted region [94, 150, 151, 153, 154].

During the inflammatory phase of cardiac healing (around the first 3 to 4 days post-MI in rodents), infiltrated neutrophils and macrophages constitute the two predominant forces eliminating dead cells and ECM debris from the infarcted area [155]. Gal-3, secreted by ECs, cardiomyocytes, and macrophages, promotes neutrophil and macrophage infiltration in infarcted myocardium [130, 154, 156]. In Gal-3-deficient mice, remarkably decreased macrophage infiltration in infarcted region was determined at 7 days post-MI along with a higher proportion of M2 phenotype [132, 152, 157]. ECM components in myocardium: laminin, fibronectin and type I collagen have been shown to bind Gal-3 and support the recruitment and retention of proteolytic M1 macrophages [158]. Sano et al. illustrated that intracellular Gal-3 played an important role in contributing to the phagocytic capacity of macrophages, whilst Caberoy et al. identified extracellular Gal-3 as a novel ligand of myeloid-epithelial-reproductive tyrosine kinase (MerTK), which induced macrophages to engulf cell debris and apoptotic cells [159, 160]. Therefore, Gal-3 tends to facilitate the clearance of cell and ECM debris, which is of great importance for the transition to reparative phase in infarcted myocardium.

The reparative phase (approximately the ensuing 4 to 14 days post-MI in rodents) is primarily characterised by infiltration and proliferation of myofibroblast, angiogenesis and subsequent scar maturation in the infarcted myocardium [155]. González et al. reported Gal-3-deficient mice exhibited worse ventricular dilation and pulmonary congestion and reduced ventricular contractile function at 7 days after LAD ligation compared to sham controls [132]. Similar outcomes at 4 weeks post-MI were also found in the study of Cassaglia et al. [157]. Higher mortalities of the Gal-3-knockout groups, with cardiac rupture as the main cause within the first week, were observed in both studies [132, 157]. In addition, significantly larger infarct size with decreased scar thickness were found in the knockout mice at 7 days after ligation, however, at 24 h and 4 weeks post-MI, similar infarct size between the knockout and wild-type groups were observed [157]. Accordingly, instead of an actual exacerbated magnitude of MI, the increase in size of infarct area at one week possibly results from severer infarct expansion caused by Gal-3 deficiency [157], which relates to consequent congestive heart failure and cardiac rupture [161]. Indeed, an in vitro study illustrated that Gal-3 was able to initiate the conversion of fibroblasts into myofibroblasts and promote their proliferation as well as the synthesis of collagen Type I [153], which is a stiff component of ECM and reinforces the tensile strength of infarct myocardium. Reportedly, either deficiency or inhibition of Gal-3 associates with attenuated collagen expression or fibrosis in the infarcted region, border zone or entire LV of non-reperfused MI models [132, 152, 156, 157, 162–165]. In addition to the reparative fibrosis, Gal-3 also promotes VEGF-mediated angiogenesis [166], which is also essentially involved in cardiac healing extending neovessels from the border zone to the infarcted region and delivering nutrients and oxygen [156]. Collectively these findings indicate a protective effect of Gal-3 in cardiac healing and remodelling during the acute phase of MI.

However, long-term overexpression of Gal-3 post-MI has been shown to correlate with exacerbated adverse remodelling and cardiac dysfunction [137]. Aldosterone was shown to induce Gal-3 expression in macrophage cells [167] and VSMC [168] resulting in vascular and cardiac fibrosis [168, 169], which implies a correlation between the renin–angiotensin–aldosterone system (RAAS) and Gal-3 expression. The intense activation of RAAS during and after MI possibly foster the overexpression of Gal-3 in global myocardium. Consistently, administration of eplerenone and spironolactone for 4 weeks after MI have been reported to downregulate Gal-3 in the infarcted myocardium of rats but have no effect on remote area [164]. Metformin also attenuated the upregulation of Gal-3 in infarcted myocardium via mitochondrial NADPH oxidase 4/PKCa/Gal-3 pathway after 4-week treatment [156]. Rabbits being treated with perindopril after coronary ligation for 4 weeks also showed downregulated myocardial and circulating Gal-3 [165].

## Galectins in metabolic disorders

Metabolic diseases have become a worldwide epidemic of the twenty-first century, with global incidence of obesity and diabetes mellitus (DM) reaching dramatic proportions. It is now widely accepted that inflammation and metabolism are two physiological processes tightly connected and regulated. Lack or alteration on their modulation can lead to metabolic complications such as the mentioned above. In fact, a chronic, low-grade inflammation has been reported to underlie obesity and DM [170, 171]. This chronic inflammatory state is characterised by abundant immune cells, particularly M1 macrophages and T cells, that infiltrate the adipose tissue and secrete increased levels of pro-inflammatory cytokines; these mediators act to perpetuate systemic inflammation and may interact with the insulin receptor and its associated downstream pathways, leading to a dysregulation in insulin response mechanisms [172, 173]. Impaired insulin signaling is compensated for the overproduction and secretion of pancreatic insulin, thus driving to insulin resistance, commonly seen in obese patients. If obesity is maintained or even aggravated, the pancreas fails to compensate for the insulin resistance, thus eventually leading to insufficient hepatic and peripheral glucose disposal and thereafter high plasma glucose levels. This is the starting point of type 2 diabetes mellitus (T2DM), which represents approximately 90% of all cases of diabetes [170]. The other 10% corresponds to type 1 diabetes mellitus (T1DM), a chronic, autoimmune disease, where immune cells, mainly T cells, attack, and destruct insulin-producing β pancreatic cells, thus leading to a defect in insulin production [174]. Notably, a long-standing hyperglycemic state can trigger production of advanced glycation end products (AGEs) that bind to organic molecules, enhancing the damage and exacerbating diabetic complications [175].

Since saccharides are crucial energy molecules in metabolic pathways, it is therefore not surprising that lectins that bind sugar groups are important modulators of metabolic homeostasis [2]. In fact, a pivotal role of some Gals in the regulation of diabetes and obesity by modulating inflammation, insulin resistance and adipogenesis has been reported [2].

### Galectin-1 and -9

In T1DM, the destruction of β pancreatic cells is mainly mediated by Th1 cells, which are regulated by Gal-1 and Gal-9. Hence, upregulation or administration of Gal-1 and Gal-9 in different animal models has been shown to significantly protect from the development of T1DM by acting on T cells [42, 176]. Remarkably, Gal-1 intraperitoneal injection not only prevented the onset of hyperglycemia, but also reversed β cell autoimmunity in the murine pancreas by reducing the amount of Th1 cells, increasing T cell-dependent IL-4 and Il-10 secretion, and causing β cell-reactive T cell peripheral deletion [42]. Gal-1 administration also accelerated the healing of pathological wounds associated with diabetes [177]. Regarding Gal-9 protective effects on T1DM, were suggested to be TIM-3 dependent [178].

Intriguingly, the role of Gal-1 in obesity and T2DM remains unclear, and controversial hypothesis have been proposed. Some groups have suggested that increased Gal-1 expression levels in serum of obese individuals may be interpreted as a compensatory mechanism to improve glucose metabolism, while many others have reported that ablation or inhibition of Gal-1 prevents adipogenesis and obesity [179]. In fact, inhibition of Gal-1 with thiodigalactoside was shown to led to weight loss in diet-induced obese mice by attenuating adipogenesis and lipogenesis and increasing expression of proteins associated with energy expenditure [180]. Another Gal-1 inhibitor, called OTX-008, was reported to strongly prevent diabetes associated-renal fibrosis [181]. The fact is that more studies are needed to further understand what cellular and molecular mechanisms link Gal-1 with the progression of metabolic disorders, because there are so many fragmentary data so far.

Gal-9 expression has been reported to increase in subcutaneous adipose tissue and in macrophages of visceral adipose tissue in obese mice, as well as in serum of T2DM patients, where it regulates disease progression [182, 183]. The suggested mechanism by which Gal-9 may regulate glucose homeostasis in T2DM is by favouring the retention of the glucose transporter GLUT-2 on the β cell surface; this transporter is expressed in pancreatic β cells and, in response to glucose stimulation, translocates to the plasma membrane, mediating the entry of glucose into the cell. Gal-9 has been shown to associate to this glucose transporter [184]. Interestingly, Gal-9 has also been revealed to attenuate the development of non-alcoholic fatty liver disease associated to obesity, by inducing apoptosis of NK cells positive for TIM3 receptor [185].

### Galectin-3

Gal-3 pro-inflammatory effects have been shown to contribute to tissue damage during diabetes and obesity. However, some conflicting results have been published. In T1DM, Gal-3 expression in DCs was shown to activate pro-inflammatory macrophages and T cells required for β-cell damage. Indeed, targeted disruption of the Gal-3 gene resulted in decreased susceptibility to multiple low dose streptozotocin-induced diabetes in mice [186]. In T2DM and obese patients, as well as in experimental animal models of obesity, circulating Gal-3 levels have been found elevated and correlate with body mass index [51, 187]. Results from animal studies have supported that Gal-3 may be involved in the onset and progression of these metabolic disorders by acting primarily at the adipose tissue level [188]. Furthermore, cardiac inflammation and lipotoxicity in obese patients has been attributed to Gal-3 pro-inflammatory properties [189]. Some studies have even suggested Gal-3 as a potential biomarker of metabolic dysregulation and cardiovascular remodelling in patients with hypertension and T2DM [190]. Intriguingly, some beneficial effects of Gal-3 have been described. Gal-3 binds AGEs, glycated proteins, and lipids during diabetes, and is considered an AGE receptor [191]. Interestingly, Gal-3 deficient mice have been shown to develop accelerated diabetic glomerulopathy compared to wild-type littermates, attributing this effect to the potential role of Gal-3 in AGE removal in the kidney [192]. However, even considering these desirable effects of Gal-3 in the diabetic kidney, its pro-inflammatory effects can equally damage the tissue [193].

In summary, the three Gals explored play pivotal roles in metabolic associated-inflammation and glucose homeostasis, thereby emerging as attractive molecules for the regulation of obesity and diabetes development. Most studies agree with a protective role of both Gal-1 and Gal-9 in T1DM, whereas some controversial data are found regarding the role of Gal-1 in obesity and T2DM. Gal-3 activity is mainly found detrimental in the progression of metabolic complications, although some studies are available attributing beneficial effects of this lectin mainly linked to AGEs removal.

## Conclusions

**Fig. 6:**
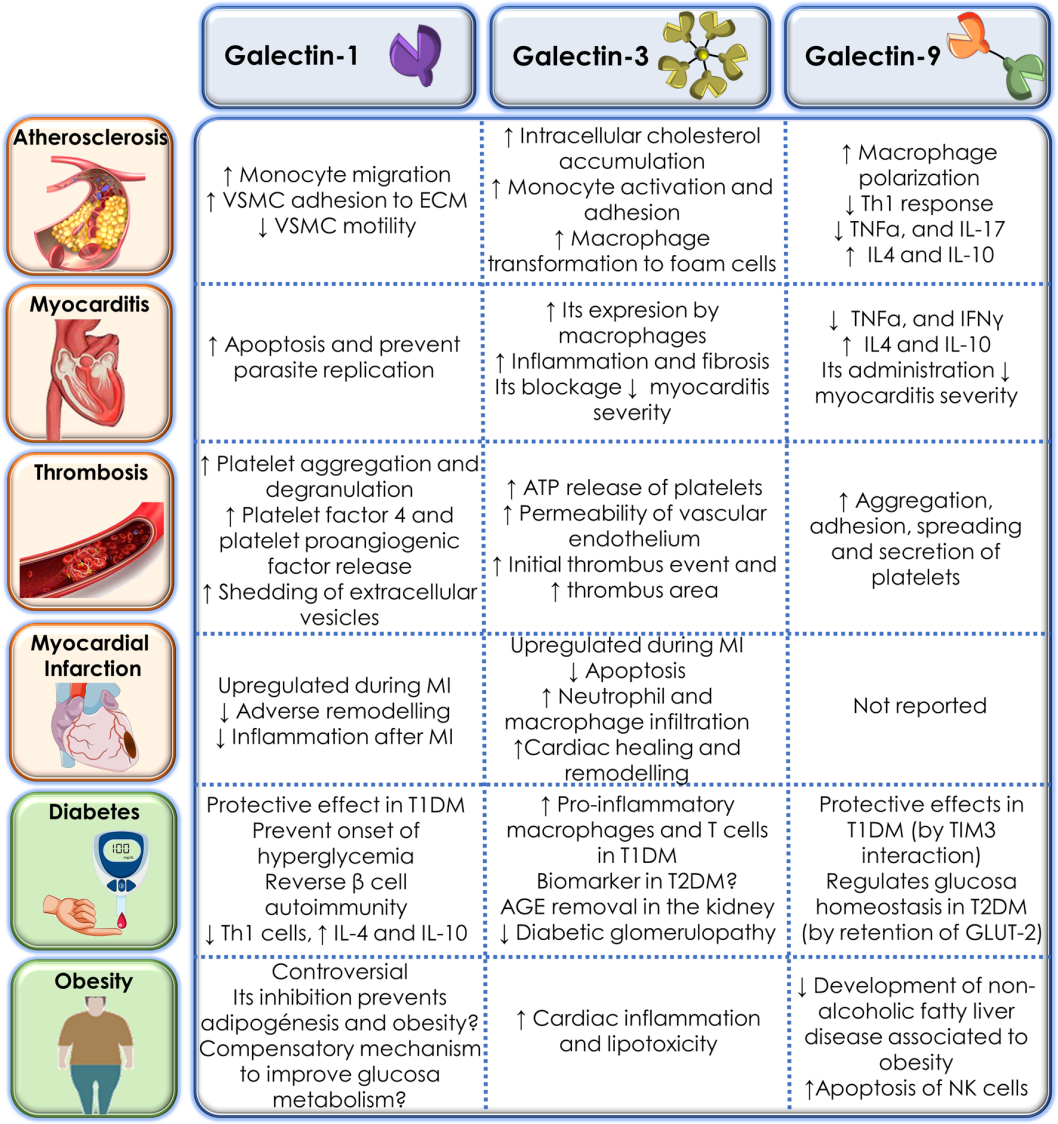
Role of Gal-1, Gal-3 and Gal-9 in cardiovascular and metabolic diseases

This review highlights Gal-1, Gal-3, and Gal-9 as essential mediators of immune-inflammatory responses, and their participation in the progression of several inflammatory driven disorders. Generally, while Gal-1 and Gal-9 seem to be mainly protective in the progression of cardiovascular and metabolic disorders, Gal-3 pro-inflammatory effects have widely been reported to aggravate disease progression (Fig. 6). However, effects attributed to each individual Gal significantly differ depending on the cell- and tissue- specific expression, as well as the pathologic context. This has important implications for the design of Gal modulatory drugs, thus potential future therapeutics may have to activate or inhibit their expression in an organ- and cell-specific manner. It is therefore evident that more studies are needed to improve our understanding how these endogenous lectins specifically work in health and disease, to consider them as appealing targets for the treatment of some highly impact human pathologies.

## Data Availability

Not applicable.

## Abbreviations

ACS: Acute coronary syndrome
AGEs: Advanced glycation end products
CRDs: Carbohydrate recognition domains
CAD: Coronary arterial disease
DAMPs: Danger-associated molecular patterns
DCs: Dendritic cells
DM: Diabetes mellitus
EAM: Experimental autoimmune myocarditis
ECs: Endothelial cells
ECM: Extracellular matrix
Gals: Galectins
GP: Glycoprotein
HUSMC: Human umbilical vascular smooth muscle cell
LAD: Left anterior descending
MCP: Modified citrus pectin
MerTK: Myeloid-epithelial-reproductive tyrosine kinase
MI: Myocardial infarction
NK: Natural killer cells
oxLDL: Oxidised low density lipoprotein
PAD: Peripheral arterial disease
PAMPs: Pathogen-associated molecular patterns
PAR4-AP: Protease-activated receptor 4–activating peptide
PMA: Phorbol myristate acetate
RAMPs: Resolution-associated molecular patterns
SLE: Systemic lupus erythematosus
TIM3: Mucin domain-containing protein 3
Treg: T regulatory
VSMCs: Vascular smooth muscle cells
VTE: Venous thromboembolism
Vwf: Von willebrand factor
